# Unreliable Yet Still Replicable: A Comment on LeBel and Paunonen (2011)

**DOI:** 10.3389/fpsyg.2015.02039

**Published:** 2016-01-13

**Authors:** Maarten De Schryver, Sean Hughes, Yves Rosseel, Jan De Houwer

**Affiliations:** ^1^Department of Experimental Clinical and Health Psychology, Ghent UniversityGhent, Belgium; ^2^Department of Data Analysis, Ghent UniversityGhent, Belgium

**Keywords:** implicit measures, reliability, power, replication

## Abstract

Lebel and Paunonen (2011) highlight that despite their importance and popularity in both theoretical and applied research, many implicit measures continue to be plagued by a persistent and troublesome issue—low reliability. In their paper, they offer a conceptual analysis of the relationship between reliability, power and replicability, and then provide a series of recommendations for researchers interested in using implicit measures in an experimental setting. At the core of their account is the idea that reliability can be equated with statistical power, such that “lower levels of reliability are associated with decreasing probabilities of detecting a statistically significant effect, given one exists in the population” (p. 573). They also take the additional step of equating reliability and replicability. In our commentary, we draw attention to the fact that there is no direct, fixed or one-to-one relation between reliability and power or replicability. More specifically, we argue that when adopting an experimental (rather than a correlational) approach, researchers strive to minimize inter-individual variation, which has a direct impact on sample based reliability estimates. We evaluate the strengths and weaknesses of the LeBel and Paunonen's recommendations and refine them where appropriate.

In their original paper, Lebel and Paunonen (2011) draw attention to a measurement revolution that has unfolded within social psychology over the past two decades and that has shaped methodological, theoretical and empirical developments outside of its borders. For much of the past century, researchers relied on a set of *direct procedures* such as semantic differential scales, feeling thermometers, and questionnaires when assessing people's attitudes, beliefs, and personality characteristics. These procedures are often deployed under the assumption that people not only have introspective access, but also the opportunity and motivation to accurately report on their psychological attributes or content. Yet it is well-known that this assumption is often violated in socially-sensitive situations (e.g., evaluations of racial, gender or religious groups), demand prone domains (e.g., job hiring or clinical assessment contexts), or instances where the individual lacks introspective access to the content under investigation (see Payne and Gawronski, 2010, for a book length treatment).

These limitations sparked a methodological revolution centered on the development and refinement of a new class of *indirect procedures*. At their core, indirect procedures seek to measure in a way that (a) circumvents a person's ability to strategically control their behavior as well as (b) captures psychological processes, attributes, or content in ways that does not depend on introspective access. A multitude of indirect procedures have now been developed and many have seen widespread application both inside and outside of psychological science, from clinical psychology (Roefs et al., 2011), to cognitive (Hahn and Gawronski, 2015), and developmental psychology (Dunham et al., 2008), as well as in neuroscience (Stanley et al., 2008), political (Nosek et al., 2010), and consumer science (Gregg and Klymowsky, 2013). The most influential of these procedures include the Implicit Association Test (IAT; Greenwald et al., 1998), evaluative priming (e.g., Fazio, 2001), and the Affective Misattribution Procedure (AMP; Payne et al., 2005; for more see Nosek et al., 2011; Gawronski and De Houwer, 2014)^1^.

Lebel and Paunonen (2011) highlight that despite their theoretical and applied implications, the vast majority of implicit measures suffer from unacceptably low levels of reliability, especially when compared to their explicit counterparts (see also Cunningham et al., 2001; Fazio and Olson, 2003; Gawronski et al., 2007). These reliability estimates (usually based on split-half correlations or coefficient alphas) range from “abysmally low (Bosson et al., 2000) to moderate (Kawakami and Dovidio, 2001)” (Lebel and Paunonen, 2011, p. 572) and are argued to have serious knock-on effects for cumulative scientific progress. In their paper, Lebel and Paunonen equate the issue of reliability with the issue of statistical power, and suggest that “lower levels of reliability are associated with decreasing probabilities of detecting a statistically significant effect, given one exists in the population” (p. 573). They also take an additional step and equate the issue of reliability with replicability. In particular, they suggest that “random measurement error, which contributes to the unreliability of measures, can prevent an experiment from being exactly repeatable” (p. 571). In other words, higher amounts of random measurement error contaminate a measure's score and decreases the likelihood that researchers will be able to replicate their own or other's findings. To put it differently, “given that the probability of replication is simply a special case of statistical power (i.e., probability of replication is the probability of detecting a statistically significant effect given one exists in the population and that the effect has already been found in at least one sample), it follows that decreasing levels of reliability should be associated with reduced likelihood of replication” (p. 573).

To test this idea, Lebel and Paunonen (2011) conducted a Monte Carlo simulation to examine the effect of different levels of reliability on the replicability of experimental findings in the context of implicit measures. The authors found that the probability of replicating an experimental effect “systematically decreased as the random measurement error contaminating the scores increased. This pattern was especially pronounced for “medium” and “large” population effect sizes and for moderate to large sample sizes (i.e., N equal to or greater than 30 per condition)” (p. 577). Based on the results of their simulation, LeBel and Paunonen put forward three main ideas. First, they argue that random measurement error should be equated with the concept of reliability—and as a result—the probability of replicating an experimental effect decreases as random measurement error (i.e., low reliability) increases. In other words, empirical results that are influenced by random measurement error cannot be replicated exactly whereas results uncontaminated by random measurement error are more likely to replicable (i.e., *probability of replication* increases as a function of *reliability*). Second, they argue that researchers should strive to improve implicit measures that suffer from unacceptable levels of reliability and gravitate toward measures known to have acceptable psychometric properties. Finally, when using implicit measures, researchers should routinely and accurately report reliability, and in the case of experimental work, provide separate reliability estimates for each and every experimental condition. The above conceptual analysis and associated recommendations certainly seem reasonable on first glance. Yet we believe that these recommendations and the assumptions they are built upon are not as straightforward as one would initially suspect. As we shall see, there is no direct or one-to-one mathematical relationship between the reliability of an implicit measure and the likelihood of replicating an experimental outcome. Random measurement error and reliability refer to two very different psychometric concepts that cannot be used interchangeably. By equating these two concepts, Lebel and Paunonen (2011) arrive at a number of conclusions that might undermine the interpretation and evaluation of data as well as the development of new procedures.

The current commentary has two main goals. First, it aims to provide a quick primer for those interested in the concept of reliability and its relation to implicit measures in experimental contexts. We recognize that this primer will likely contain statistical and psychometric concepts (reliability, power and replicability) that some readers are already familiar with. Our aim is to demonstrate when these concepts are *combined*, a number of conclusions emerge that are, at first sight, counter-intuitive, especially for researchers who are less familiar with psychometric theory and who merely employ implicit measures as tools in their experimental work. Second, LeBel and Paunonen made several recommendations for the experimental use of implicit measures. Like any recommendations, these have the potential to influence the actions of editors and reviewers, as well as the activities of the researcher. We therefore aim to evaluate the strengths and weaknesses of these recommendations, and refine them where appropriate.

## The relationship between reliability and replicability

At the core of LeBel and Paunonen's paper is the notion that reliability is intimately connected with the concepts of statistical power and replicability. To support this assertion, they point to a number of publications demonstrating a positive relationship between the reliability of a dependent variable and the statistical power needed to observe differences between experimental groups or conditions where such differences exist (Sutcliffe, 1958; Rogers and Hopkins, 1988). Yet contrary to their suggestions, the relationship between reliability and statistical power is not a simple, positive or direct one (see Overall and Woodward, 1975, 1976; Fleiss, 1976; Nicewander and Price, 1978; Hopkins and Hopkins, 1979; Williams and Zimmerman, 1981; Overall and Ashby, 1991; Williams et al., 1995). For nearly 50 years, the link between reliability and power has been debated in the psychometric literature, with several authors suggesting a positive relation between these two concepts (e.g., Sutcliffe, 1958; Rogers and Hopkins, 1988) while others argue for the very opposite (negative) relationship (e.g., Overall and Woodward, 1975, 1976; Nicewander and Price, 1978). Thus, despite suggestions to the contrary, there appears to be a paradox in arguing for a general or fixed mathematical relation between reliability and power (for more see Williams et al., 1995).

This has serious implications for Lebel and Paunonen's (2011) original argument. If there is no fixed relationship between reliability and power, and if replicability is “simply a special case of statistical power” (p. 573), then it follows that there is no general or fixed relation between reliability and replicability. A simple demonstration might help to illustrate our point more clearly. In their original paper, LeBel and Paunonen ran a Monte Carlo simulation to examine the impact of unreliability in a dependent variable on the replicability of results for a simple two-group between-subjects test of means. This simulation revealed that the probability of replicating an experimental effect systematically decreased as the random measurement error contaminating the scores increased. We set out to replicate these findings, but instead of using simulations, we arrived at an exact solution via the formula for calculating power for a two-sample *t*-test with equal variances (i.e., $\sigma_{1}^{2}=\sigma_{2}^{2}=\sigma^{2}$). Working through this example will illustrate the paradox of equating reliability with power or replicability.

First, let X denote observed scores, which can be defined as the sum of unobserved true-scores (*T*) and error-scores (*E*). Now, following classical test theory, we can define reliability as the ratio of true-score variance to observed-score variance, $\rho {\;}_{XX^{\prime }=\sigma_{T}^{2}/\sigma_{X}^{2}}$, with *X* = *T* + *E*, or, we can define reliability in terms of true- and error-score variances, $\rho {\;}_{XX^{\prime }}=1-\frac{\sigma_{E}^{2}}{\sigma_{T}^{2}+\sigma_{E}^{2}}$ and $\sigma_{X}^{2}=\sigma_{T}^{2}+\sigma_{E}^{2}$ (Lord and Novick, 1968). Let *N* be the number of observation in each condition, δ the smallest relevant difference or effect size and δ > 0. Then for a given alpha (α), the power π(δ) can be calculated as follows:

$$\begin{array}{l} \pi \left(\delta \right)=1-F_{N-1,\frac{\sqrt{N}\delta }{\sigma }}\left(t_{N-1,\alpha }\right), \end{array}$$

where *F* is the cumulative distribution function of the non-central t-distribution, with *N* − 1 degrees of freedom and with non-centrality parameter $\sqrt{\text{N}}\;\delta ∕\sigma$.

**Figure 1:**
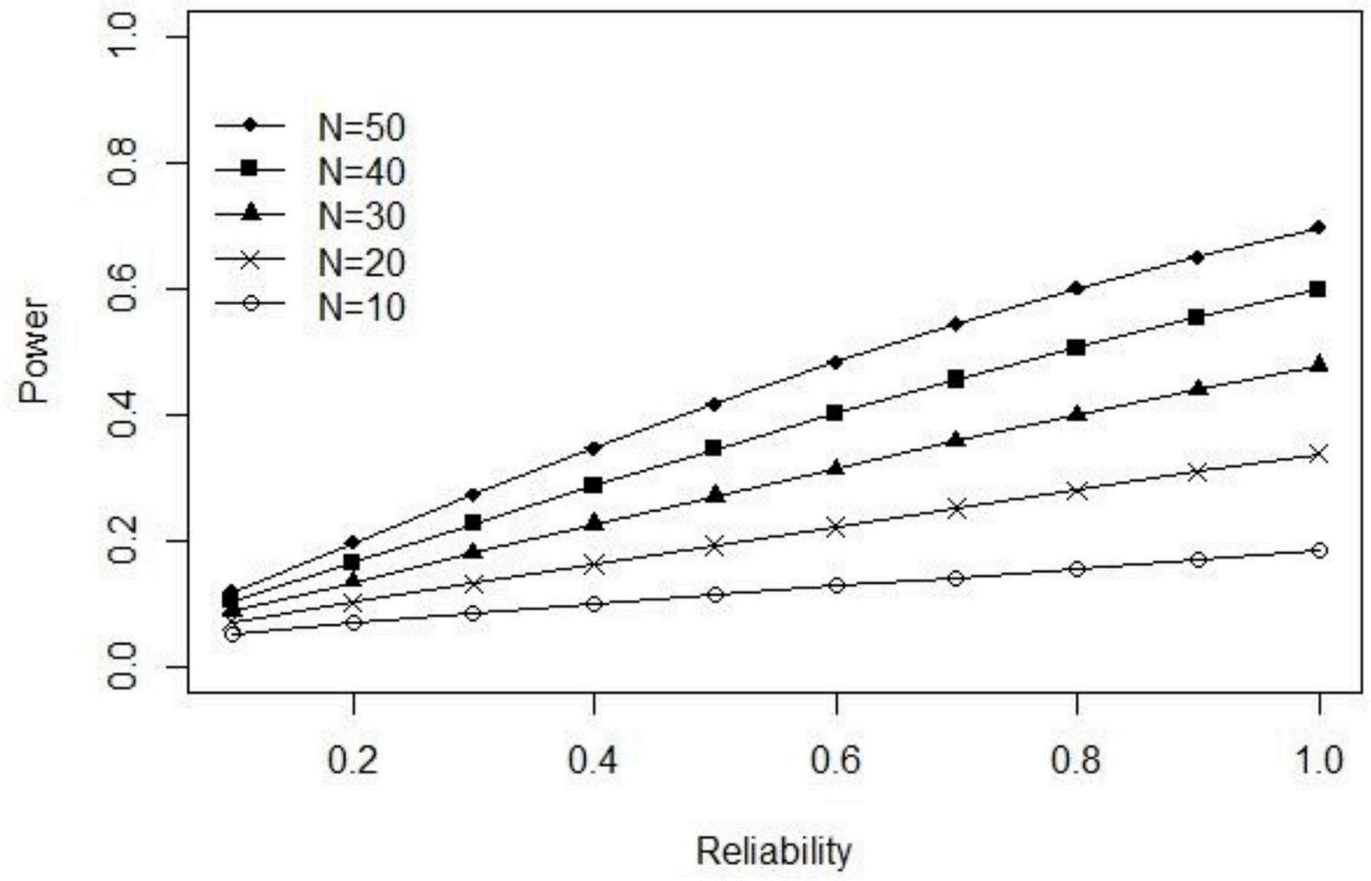
**Exact power as a function of reliability when true-score variance is fixed at 1.00**.

In their original Monte Carlo simulation, Lebel and Paunonen (2011) fixed the true-score variance $\left(\sigma_{T}^{2}\right)$ at 1.00 while allowing the error-score variance ($\sigma_{E}^{2}$) to vary in order to guarantee *a priori* levels of reliability (i.e., $\sigma_{E}^{2}=\left(1-\rho_{XX}\right)∕\rho_{XX}$). Consequently, the observed score variance (σ^2^) used in the above power function can be expressed as ($\sigma^{2}=\sigma_{X}^{2}=1.00+\left(1-\rho_{XX}\right)∕\rho_{XX}$). The pattern of results obtained from our power formula for ρ_*XX*_ ∈ {0.10, 0.20, …, 1.00}, *N* ∈ {10, 20, …, 50}, α = 0.05, and δ = 0.50, can be observed in Figure 1. When true-score variance is fixed, our power function reveals an almost identical (positive) relation between power and reliability as seen in the author's original paper.

**Figure 2:**
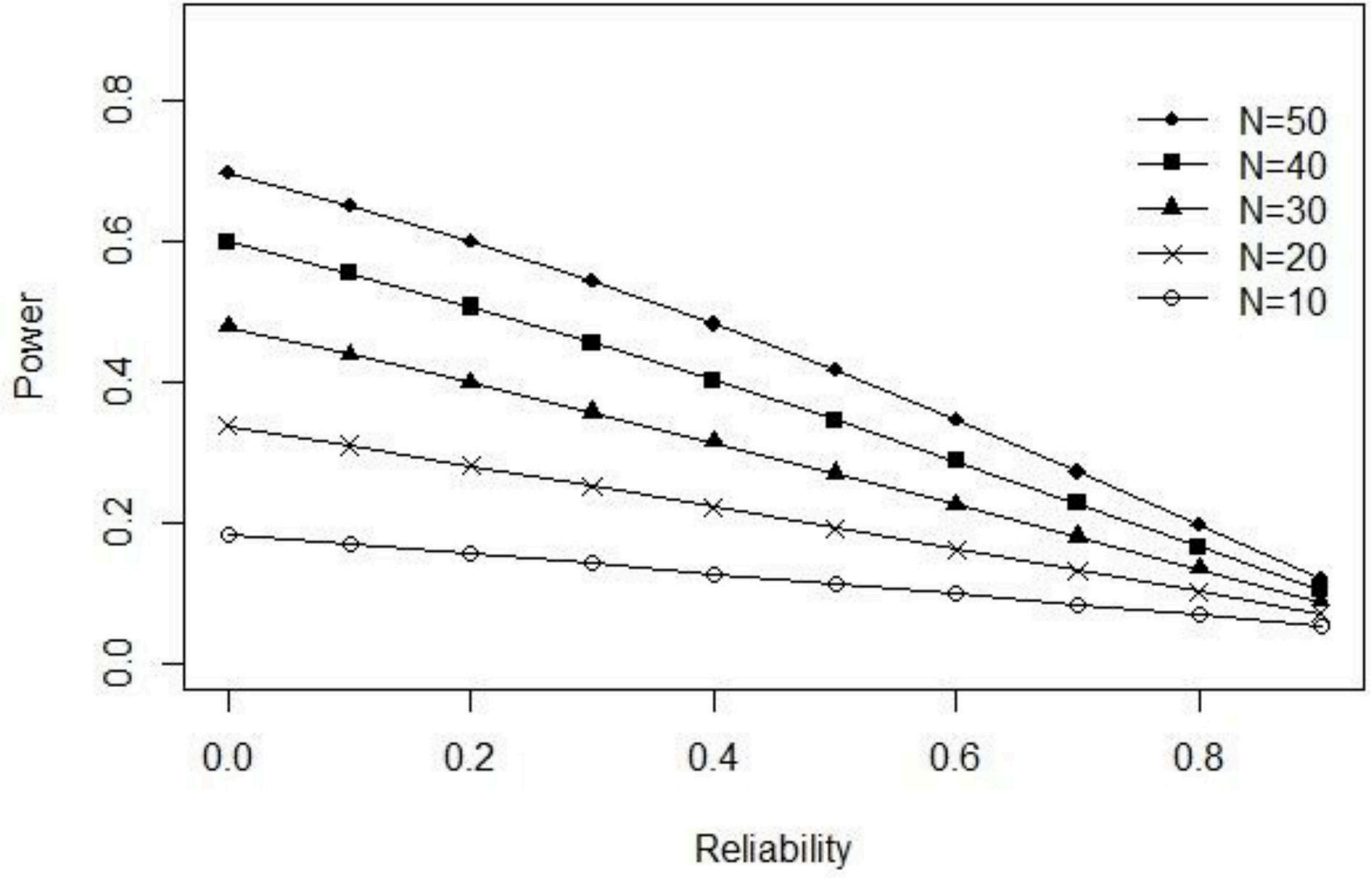
**Exact power as a function of reliability when error-score variance is fixed at 1.00**.

Now imagine that instead of true-score variance we fix error-score variance ($\sigma_{E}^{2}$) at 1.00 and allow the true-score variance to vary as a function of different levels of reliability. In this case the true-score variance as a function of reliability is ($\sigma_{T}^{2}=\frac{1.00}{1-\rho_{XX}}-1.00$). The observed score variance can then be expressed as ($\sigma^{2}=\sigma_{X}^{2}=\frac{1.00}{1-\rho_{XX}}$). The pattern of results obtained from our power formula for (ρ_*XX*_ ∈ {0.00, 0.10, …, 0.90}, ∈ {10, 20, …, 50}, α = 0.05, and δ = 0.50), can be observed in Figure 2. When error-score variance is fixed, our power function reveals an entirely opposite (negative) relationship between power and reliability as compared to that reported by Lebel and Paunonen (2011).

The above example clearly illustrates the paradox of equating reliability with power or replicability. Consequently, Lebel and Paunonen (2011) do not provide sufficient information to disentangle the various relationships that can potentially exist between reliability and replicability in their original paper. Instead they simply focus on the first of these possibilities (i.e., fixed true-score variance) and thus their conclusions should not be overgeneralized and only applied to such situations.

## Evaluating research findings characterized by low levels of reliability

If it is the case that there is no fixed mathematical relation between reliability and power, then LeBel and Paunonen's second recommendation also needs to be re-examined (i.e., that researchers should “improve those implicit measures having unacceptable levels of reliability or then utilize implicit measures known to have acceptable psychometric properties”). To illustrate this more clearly, imagine that you are a social psychologist interested in understanding how humans come to like and dislike novel stimuli. You begin by formulating a relatively simple hypothesis that evaluative responses to stimuli can be changed by providing people with verbal information about that stimulus. To test this hypothesis, you provide a group of thirty participants with a set of attitude-relevant instructions (e.g., “*Luupites are good and Niffites are bad*”) and another group of thirty participants with attitude-irrelevant instructions (e.g., the basic steps required to waltz at a party). Thereafter, you administer a test of automatic evaluative responding such as an IAT wherein participants have to categorize items related to Luupites and positive words using one response key and items related to Niffites and negative words using another response key. In a second block of trials these response assignments are reversed so that Luupite-related items and negative words are assigned to the first key while Niffite-related items and positive words are assigned to the second key. The difference in performance during the first relative to the second phase (known as the IAT effect) is considered to provide an overall measure of how readily people prefer Luupites compared to Niffites (see De Houwer, 2006; Gregg et al., 2006, for studies along these lines).

**Figure 3:**
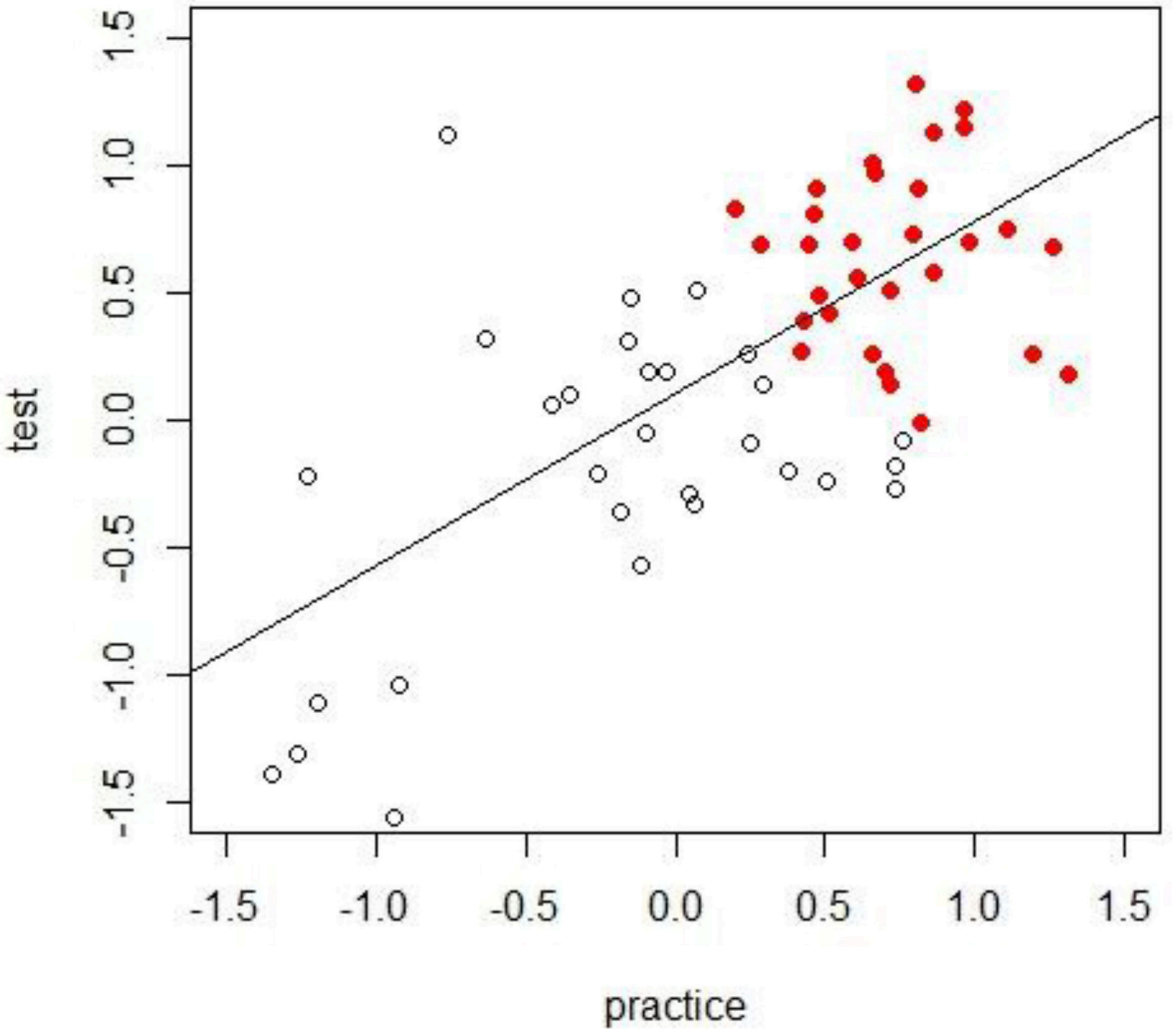
**IAT-scores for the practice and test trials for attitude-relevant (filled circles) and attitude-irrelevant instruction (white circles) conditions in our hypothetical example**. Note that both practice blocks and test blocks are taken into account for estimating the final IAT-score.

Now imagine that data collection is finished. You create a scatterplot and regression line using the IAT scores obtained from the test trials and practice trials for participants in the two instruction conditions (see Figure 3). Analyses reveal that participants provided with attitude-irrelevant instructions displayed a non-significant preference for Niffites over Luupites (*M* = −0.25, *SD* = 0.55) while participants provided with attitude-relevant instructions display a clear evaluative bias for Luupites over Niffites (*M* = 0.68, *SD* = 0.22). Running a *t*-test with a Welch's correction reveals a significant difference between the mean preferences of the two experimental conditions, *t*_(38.11)_ = 8.54, *p* < 0.001.

In-line with Lebel and Paunonen's (2011) recommendations, you then estimate the reliability coefficient for both groups using a bootstrap procedure, wherein 1000 random-splits are drawn from the data. For each random split, you estimate a correlation between one split and another. This yields a final reliability estimate in the form of a Spearman-Brown corrected mean split-half correlation. Somewhat surprisingly, you observe a higher reliability estimate for IAT scores in the attitude-irrelevant group (mean *r* = 0.92) compared to those in the attitude-relevant group (mean *r* = 0.57). The fact that (a) the scores of these two experimental conditions vary in their reliability estimates and (b) the reliability estimate obtained in the attitude-relevant condition is rather low, may cause you as an experimenter, and the individual reviewing your paper, some concern. But is this concern really justified?

The low reliability estimate observed in the attitude-relevant condition tells us that, in this case, *local* measurement precision (due to range restriction in the observed scores) is relatively poor: the relative ordering of participants in this group would probably change if the test was administered under similar contextual conditions. Put another way, we have a relatively homogenous group with respect to the underlying evaluation and our test is not capable of capturing individual differences *within* that group. But note that this was not the original aim of our study (for more on this point see below). What is important to appreciate here is that the lower level of reliability in the attitude-relevant compared to irrelevant condition does not necessarily imply a higher level of measurement error: if we estimate the group observed-score variances for the attitude-irrelevant ($\sigma_{X1}^{2}=0.31$) and attitude-relevant instructions conditions ($\sigma_{X2}^{2}=0.05$) and input these values into the reliability formula $\left(\sigma_{E}^{2}=\sigma_{X}^{2}-\rho_{XX^{\prime }}\sigma_{X}^{2}\right)$, then the estimated group error-score variance of the attitude-irrelevant group $\left(\sigma_{E1}^{2}=0.31-0.92*0.31=0.025\right)$ appears to be slightly larger than that of the attitude-relevant group $\left(\sigma_{E2}^{2}=0.05-0.57*0.05=0.022\right)$. In other words, individual IAT-effects in the attitude-relevant group were estimated with a similar level of precision as in the attitude-irrelevant group. The difference in reliability estimates are therefore heavily influenced by differences in true-score variances.

So is it problematic that we observed a rather low reliability score in the attitude relevant condition? The answer—like many in psychological science—is that it depends. Low reliability scores are problematic only if we were interested in differences between individuals (within a group) rather than between groups. Yet in typical experimental designs, including those that use implicit measures, researchers prefer homogenous groups. That is, they strive to decrease observed score-variances within groups or conditions in order to reduce the impact of individual differences, which usually translates into lower true-score variances (Nicewander and Price, 1978, p. 407). Such strategies tend to decrease the residual variance in statistical tests such as *t*-tests or ANOVAs, and as we discussed previously, this often results in lower reliability estimates whenever error-score variances are held constant (also see Williams et al., 1995). Of course, researchers can always improve their measure by replacing their existing test with a tau-equivalent alternative, that is, a comparable test with similar true- but lower error-scores. Doing so will not only lead to a more reliable test, but, due to fixed true-score variance, a more powerful test (as was the case with LeBel and Paunonen original simulation study; see Nicewander and Price, 1978). However, by replacing one measure with another in situations where their true-scores do not correlate perfectly, researchers introduce uncertainty about the underlying construct in question. Therefore the claim that “researchers need to improve those implicit measures having unacceptable levels of reliability or utilize implicit measures known to have acceptable psychometric properties” should be interpreted with caution.

What about the fact that the reliability estimate in the attitude relevant condition was lower than that in the irrelevant instructions condition? Although Lebel and Paunonen (2011) argue that “differences in observed scores across groups cannot be meaningfully interpreted” in situations where “reliability is drastically different across conditions” (p. 580), we argue that even in such cases groups *can* be meaningfully compared, so long as differences in reliability estimates are primarily due to differences in true-score rather than error-score variance (see DeShon, 2004). Thus, in the current example (where error-scores were similar), applying a *t*-test using Welch's correction will be robust enough to test hypotheses about meaningful mean group differences even though those groups differed in their respective reliability estimates^2^.

In short, LeBel and Paunonen's second recommendation should be interpreted with care. The take home message here is that researchers and reviewers should both be aware that low levels of reliability are not necessarily due to increased levels of error-score variance but can also be due to decreased levels of true-score variance. Likewise, the authors' suggestion that some researchers “have been able to easily replicate effects using certain implicit measures, despite their low reliability” (p. 579) might reflect the fact that low reliability is sometimes due to reduced true-score variance rather than increased error-score variance. Therefore, should researchers try to increase the reliability of implicit measures? On the one hand, we believe that low reliability is acceptable when it occurs due to a reduction in true-score variance. On the other hand, researchers can always improve their (implicit) measure by reducing error as long as this reduction does not affect the variance that is due to the construct of interest. But only by conducting a thorough analysis of different sources of variance can we disentangle these various possibilities.

## Should reliability estimates be reported separately for each experimental condition?

Finally, we agree with the authors that “evaluating (and reporting) the reliability of scores produced by an implicit measure should be viewed as a mandatory requirement when gauging the robustness of a finding” along with the evaluation of sample size, *p*-values, and confidence intervals.” Yet for the reasons noted above, reporting reliability estimates without also providing at least the mean scores and standard deviations of the samples (which would allow the reader to infer true—and error-score variance^3^) is off little value. Moreover, we do not agree that “reliability estimates should be reported separately for each experimental condition,” except for situations where the researcher is interested in individual differences within the sample of a particular condition. We are thus somewhat surprised by the example given by LeBel and Paunonen to motivate their argument (see Figure 5, p. 580). The authors describe a hypothetical experiment with a control and treatment group that is not unlike our own example above. It is reasonable to assume that (a) these two groups do not initially differ with respect to the underlying attribute of interest or other task-relevant factors such as demographics, (b) do differ after the intended manipulation and that (c) this difference can be observed in their respective implicit test scores. Based on the reliability index of the entire sample (α = 0.70) and the scatterplot provided by the authors on p. 580, their test seems to be a *reliable* and *valid* measure of the underlying attribute. Surprisingly, however, the authors conclude that this reliability index is “artificially inflated due to group mean differences and is completely erroneous” (p. 580). They base this conclusion on the reliability estimates obtained from each of the experimental conditions (both *r* < 0.07), both of which lack internal consistency.

This interpretation seems problematic. In this and other between-groups experiments, the researcher is not interested in examining individual differences within either the control or treatment group. Rather, they are interested in the extent to which individuals from these two groups differ from one another and often use a summary measure (e.g., mean) to do so. Therefore, it seems a little strange to evaluate the implicit measure based on its capacity to detect individual difference within each of the two groups. The reliability estimate for the entire sample and the scatterplot do indicate that the test is capable of detecting differences in the entire sample. Instead of being “artificial” in nature, those differences appear to be due to the intended manipulation and this is illustrated by the fact that there is only a shift in location between the two observed distributions. In other words, the test is doing precisely what the researchers selected it to do. Even if reliability scores were low within, or differed between experimental conditions, this would not be a problem provided that—as we mentioned above—the difference in those reliability estimates was mainly due to differences in true- rather than error-score variance.

In short, the above example seems to be inconsistent with the authors' recommendations. On the one hand, they suggest that researchers “must rule out factors that can reduce the accuracy of reliability estimates, such as the restriction of range …(p. 578)” whenever they want to evaluate the reliability of an implicit measure. On the other hand, they suggest that a reliability estimate be calculated for each experimental condition. But this latter suggestion will likely involve reliability estimates that are calculated from a restricted range of scores—a direct contradiction of what the authors recommend above. As we previously mentioned, experimental research typically involves the creation of homogenous groups. A consequence of this is that the range of scores obtained from those groups will likely be *restricted* and thus are not representative of those that would be obtained from a sample representing the entire population.

## Discussion

As Cronbach (1957) eloquently stated “the job of science is to ask questions of Nature” (p. 671), and in psychology, these questions have traditionally been asked and answered in two different ways. On the one hand, the correlational approach strives to maximize inter-individual variation in order to explore the relationship between those differences and the phenomenon of interest (i.e., there is a preference for heterogeneous samples). This may be in the service of explaining or predicting when those differences will lead to one outcome vs. another. In such a context, the researcher is often interested in maximizing true-score variance so that the test-scores of different individuals can be meaningfully interpreted. On the other hand, the experimental approach strives to minimize inter-individual variation in order to explore the impact of a particular manipulation on the group as a whole or sub-samples within that group (i.e., there is a preference for homogenous samples). This is often to test causal hypotheses and to make confident causal assumptions about the relationship between one event and another. In such a context, the researcher is typically interested in minimizing true-score variance within conditions so that tests-scores reflect the impact of the intended manipulation rather than erroneous confounds. Thus depending on the scientist's goals and values, the same (implicit) measure may be characterized as either reliable or unreliable as a function of how that researcher responds to true-score variance. High reliability is typically preferable for the correlator while (ironically) the opposite is true for the experimenter because this will lead to a more powerful test. Paradoxically, Lebel and Paunonen (2011) argue that in order to obtain a more powerful test experimenters should strive to develop and use more reliable (implicit) measures.

Yet the paradox for the experimenter is that high observed reliability sometimes leads to more powerful tests and at other times leads to less powerful tests and this makes any discussion about fixed, direct or one-to-one relations between reliability and power or replicability seemingly problematic. On the one hand, we agree with the authors that when true-score variance is fixed an increase in error-score variance will decrease the reliability of a test—and by implication—the likelihood of replication. However, focusing attention on this situation results in an overly simplified view of how reliability relates to replicability that is fraught with conceptual danger (see Nicewander and Price, 1978; Williams et al., 1995 for related arguments). For instance, our own analyses show that it is possible to increase the power of a statistical test (and by implication the likelihood of replication) by decreasing the reliability of an (implicit) measure (e.g., by using more homogeneous samples). It is also the case that implicit measures characterized by low levels of reliability are not necessarily problematic so long as that reliability is a function of reduced true-score variance. Moreover, if researchers aim to explore the reliability of different experimental conditions and report them separately, then low reliability estimates might very well be expected, and even desired. In this case the reliability estimate for the entire sample is not “artificial” but meaningful insofar as it tells us that the measure is capable of detecting individual differences given the range of the true-scores.

Of course we have largely focused on differences in true-score variances throughout our commentary in order to reinforce our central message. Nevertheless, we fully acknowledge that reliability also depends on the amount of error-score variance and that both correlator and experimenter should strive to minimize the impact of this factor where possible. Perugini et al. (2010) discuss some useful strategies (e.g., using standardized instructions, presenting stimuli in an identical order across participants) that can reduce error-score variance without affecting true-score variance. Also, more advanced psychometric models could be applied to disentangle content specific variance (i.e., true-score variance) from method specific variance (i.e., systematic error-score variance that might influence the true-score variance). For instance, it is well-known that measures inferred from raw reaction times can be confounded by general response speed (Fazio, 1990; Faust et al., 1999). By scaling these measures by units of standard deviations, the reliability and validity of these measures can be increased (Greenwald et al., 2003; Mierke and Klauer, 2003). Our point is simply that efforts to control error (both random and systematic) will always be important and impact the reliability of an implicit measure in a positive way. But researchers cannot simply equate the former with the latter as Lebel and Paunonen (2011) suggest. Instead, researchers should be aware that low reliability is not always a problem of random measurement error - and in some instances—might actually reflect tight experimental control.

## Author contributions

MDS wrote the first draft. MDS, SH, YR, and JDH wrote subsequent drafts. MDS and YR, ran the power study.

## Funding

This research was supported by grant BOF09/01M00209 of Ghent University to JDH.

### Conflict of interest statement

The authors declare that the research was conducted in the absence of any commercial or financial relationships that could be construed as a potential conflict of interest.

## Footnotes

^1^In-line with De Houwer (2006) we define a procedure as “direct” or “indirect” based on the way in which the measurement context is arranged to capture the behavior of interest (e.g., verbal, speeded categorization of stimuli). We also define the outcome derived from direct procedures as an “explicit” measure, and the outcome derived from indirect procedures as an “implicit” measure based on the properties of the psychological attribute under investigation. Put simply, “implicit” and “explicit” refer to the operating conditions under which a psychological attribute influences measurement outcomes rather than the procedure itself.

^2^For comparing more than two means, a non-parametric tests or Bayesian ANOVA can be applied (see Kruschke, 2014), although it should be noted that ANOVAs are rather robust against heteroscedasticity.

^3^Note that true-score variance can still be influenced by method specific variance (systematic error). More advanced psychometric models could be used to disentangle content specific variance and method specific variance.
